# Therapeutic effects of methylphenidate for attention-deficit/hyperactivity disorder in children with borderline intellectual functioning or intellectual disability: A systematic review and meta-analysis

**DOI:** 10.1038/s41598-019-52205-6

**Published:** 2019-11-04

**Authors:** Cheuk-Kwan Sun, Ping-Tao Tseng, Ching-Kuan Wu, Dian-Jeng Li, Tien-Yu Chen, Brendon Stubbs, Andre F Carvalho, Yen-Wen Chen, Pao-Yen Lin, Yu-Shian Cheng, Ming-Kung Wu

**Affiliations:** 1 Department of Emergency Medicine, E-Da Hospital, Kaohsiung, Taiwan; 2 School of Medicine, College of Medicine, I-Shou University, Kaohsiung, Taiwan; 3 WinShine Clinics in Specialty of Psychiatry, Kaohsiung, Taiwan; 4 Department of Psychiatry, Tsyr-Huey Mental Hospital, Kaohsiung Jen-Ai’s Home, Kaohsiung, Taiwan; 5 Graduate Institute of Medicine, College of Medicine, Kaohsiung Medical University, Kaohsiung, Taiwan; 6 Department of Addiction Science, Kaohsiung Municipal Kai-Syuan Psychiatric Hospital, Kaohsiung, Taiwan; 7 Department of Psychiatry, Tri-Service General Hospital; School of Medicine, National Defense Medical Center, Taipei, Taiwan; 8 Institute of Brain Science, National Yang-Ming University, Taipei, Taiwan; 9 Physiotherapy Department, South London and Maudsley NHS Foundation Trust, London, UK; 10 Department of Psychological Medicine, Institute of Psychiatry, Psychology and Neuroscience (IoPPN), King’s College London, De Crespigny Park, London, UK; 11 Faculty of Health, Social Care and Education, Anglia Ruskin University, Chelmsford, UK; 12 Department of Psychiatry, University of Toronto, Toronto, ON Canada; 13 Centre for Addiction & Mental Health (CAMH), Toronto, ON Canada; 14 Prospect Clinic for Otorhinolaryngology & Neurology, Kaohsiung, Taiwan; 15 Department of Psychiatry, Kaohsiung Chang Gung Memorial Hospital and Chang Gung University College of Medicine, Kaohsiung, Taiwan; 16 Institute for Translational Research in Biomedical Sciences, Kaohsiung Chang Gung Memorial Hospital, Kaohsiung, Taiwan

**Keywords:** Intelligence, ADHD

## Abstract

Attention-deficit/hyperactivity disorder (ADHD) frequently co-occurs with intellectual disability in children, and may further compromise learning. Methylphenidate is a first-line treatment for ADHD, however no previous meta-analysis has evaluated its overall efficacy for ADHD in children with comorbid intellectual disability (ID) or borderline intellectual functioning. The PubMed/MEDLINE, Cochrane CENTRAL and ScienceDirect databases were systematically searched from inception through 2018/7/15 for clinical studies that investigated the effects of methylphenidate in children with ADHD and ID. A random-effects model meta-analysis was used for data synthesis. Eight studies (average Jadad score = 2.5) enrolling 242 participants receiving methylphenidate and 181 participants receiving placebo were included. The meta-analysis showed that methylphenidate led to a significant improvement in ADHD symptoms relative to placebo (Hedges’ *g* = 0.878, *p* < 0.001). Meta-regression analysis pointed to an association between the dose of methylphenidate and overall improvement in ADHD severity (slope = 1.334, *p* < 0.001). Finally, there was no significant difference in drop-out rate [odds ratio (OR) = 1.679, *p* = 0.260] or rate of treatment discontinuation due to adverse events (OR = 4.815, *p* = 0.053) between subjects receiving methylphenidate and those taking placebos. Our study suggests that methylphenidate retains its efficacy in children with ADHD and borderline intellectual functioning or ID.

## Introduction

Attention-deficit/hyperactivity disorder (ADHD) can present with symptoms encompassing inattentive, hyperactivity and impulsivity domains, and is associated with significant psychosocial and academic dysfunctions^1^. ADHD is the most common neurobehavioral disorder amongst children and adolescents^2^. In addition, children with probable ADHD have been reported to have a 16-point decrease in IQ compared to those without ADHD^3^. These findings highlight an association between ADHD and subaverage intelligence^4^. Both pharmacological and non-pharmacological treatments have shown efficacy for the management of ADHD^2,5^.

Among pharmacological treatments, methylphenidate has been recommended as a first-line agent for ADHD^6^. It primarily acts through the inhibition of presynaptic dopamine and to a lesser extent norepinephrine transporter, thereby increasing the concentration of monoamines in the synaptic cleft^7^. Adjusting the dose on an individual basis has been reported to be crucial for optimizing behavioral improvement^8^. In addition, the beneficial effects of methylphenidate for the management of ADHD have been well documented in review articles^9^, pairwise meta-analyses^10^, and network meta-analyses^11^.

Despite the possible association between ADHD and intellectual disability (ID), most treatment trials for ADHD have excluded children with ID or borderline intellectual functioning, partly because children with ID often have coexisting medical problems and are also less likely to self-report adverse events^4^. The reliability of data derived from previous studies on the therapeutic effectiveness of methylphenidate in children with ID or borderline intellectual functioning is further hampered by crossover designs, short trial durations, differences in dosing regimens, and small sample sizes^4^. Whilst some studies have demonstrated a poorer therapeutic response in children with ID or borderline intellectual functioning compared to those without^12^, other studies have not found a significant difference in efficacy between the two groups^4^. In addition, a dose-dependent therapeutic effect was reported in one study^13^ but not in others^14,15^. Furthermore, one report failed to demonstrate an overall therapeutic effect for methylphenidate^16^ in this patient population. There are also inconsistencies in the therapeutic effects of methylphenidate on the conduct subscale. While some studies have demonstrated positive outcomes^17,18^, others have failed to show any beneficial impact^14,15^. Moreover, most studies have failed to show that methylphenidate significantly improved cognitive performance as measured by a continuous performance test (CPT)^14,16,18^. A recent systematic review of different RCTs reported that people with ID had a lower (40–50%) response rate to methylphenidate than those without ID^19^. However, the authors did not conduct meta-analysis to reinforce their conclusion. Due to these gaps in the literature, the aim of this systematic review and meta-analysis of randomized controlled trials was to assess the therapeutic effects of methylphenidate in children with ADHD and co-occurring ID or borderline intellectual functioning.

## Materials and Methods

### Guidelines and protocol

This systematic review and meta-analysis was conducted according to the guidelines presented in the *Preferred Reporting Items for Systematic Reviews and Meta-Analyses* (PRISMA) statement^20^ (Supplementary Table 1, available online). An *a priori* defined but unpublished protocol (available upon request to the authors) that was approved by the Institutional Review Board of Tri-Service General Hospital (TSGHIRB: B-105-12) was followed.

### Search strategy and identification of eligible studies

Two investigators (YS Cheng and CK Sun) independently searched the PubMed/MEDLINE, Cochrane Library and Science Direct electronic databases from inception to 2018/7/15, using the following keywords: (Stimulants or methylphenidate) and (mental retardation or intellectual disability or development delay or mental handicap or borderline intellectual functioning). We limited the results to clinical trials. In addition, the ClinicalTrials.gov database was searched using the following keywords: (ADHD and methylphenidate). This search strategy was augmented through a hand search of reference lists for eligible articles as well as relevant clinical guidelines and review articles^2,7,10,21^.

Two authors (YS Cheng and CK Sun) screened the titles and abstracts of retrieved references for eligibility. A list of potentially eligible studies was constructed by consensus, after which full-text examinations were conducted. A third reviewer (PT Tseng) was consulted if any inconsistencies arose.

### Eligibility criteria

The inclusion criteria were as follows: (1) peer-reviewed articles investigating the efficacy of methylphenidate on behavioral symptoms in children and adolescents with borderline intellectual functioning or ID symptoms who were experiencing behavioral problems related to ADHD according to the diagnostic criteria in the International Statistical Classification of Diseases and Related Health Problems (ICD), in the Diagnostic and Statistical Manual of Mental Disorders (DSM) or other standardized rating scales; and (2) articles that were randomized placebo-controlled trials conducted in humans. No language restrictions were applied.

The exclusion criteria were as follows: (1) animal studies; (2) trials not related to the treatment effect of methylphenidate on behavioral symptoms related to ADHD; and (3) studies without a placebo group (i.e., head-to-head trials).

### Methodological quality appraisal

The methodological quality of the included studies was evaluated using the Jadad scale^22^. Jadad scores were calculated for each included study and encompassed three aspects of study quality, namely, randomization, blindness, and attrition rates. Scores ranged from zero (poor quality) to five (high quality).

### Outcome assessment

For comprehensive evaluation, we studied the association between changes in clinical symptoms of ADHD and use of methylphenidate at two stages. Initially, we evaluated the change in overall severity of ADHD symptoms, which was defined as the primary outcome. At the second stage, to provide more information for clinicians, we assessed the changes in severity of ADHD symptoms on different subscales, which were defined as the secondary outcomes.

The secondary outcomes of interest included any rating scales that evaluated the three main categories of behavioral problems in ADHD, including conduct behavior, hyperactivity and inattentive symptoms. We also evaluated differences in CPT results between groups treated with placebo and those treated with methylphenidate. Finally, tolerability as assessed by the drop-out rate and the rate of treatment discontinuation due to adverse effects was also analyzed.

### Data extraction and management

Two independent authors extracted data from the eligible studies into a database of pre-determined variables of interest. The extracted variables included: changes in the severity of ADHD symptoms, mean age (years), female proportion, mean body mass index (BMI), methylphenidate treatment duration (weeks), and methylphenidate dosage (mg/kg/day).

When data were not available in the articles, we tried to contact the corresponding authors by email to request additional data on at least two different occasions one week apart.

### Statistical analysis

Due to the presumed heterogeneity among the included studies, data were analyzed using random-effects meta-analysis models rather than fixed effects models^23^ on the Comprehensive Meta-Analysis software version 3 platform (Biostat, Englewood, NJ). Effect sizes (ESs) of changes in ADHD symptoms between groups were analyzed using Hedges’ *g* and 95% confidence intervals (95% CIs). For the secondary outcomes, because all the input-data were based on the same rating scale, we further calculated the mean differences (MDs), which could provide clearer and more relevant information for clinicians. In the situations of dichotomous outcome, the ESs were analyzed using odds ratio (OR) and 95% CIs.

Heterogeneity was evaluated using the Q statistic^24^, and the *I*^2^ statistic was used to evaluate the proportion of variation^25^. We examined publication bias by visually inspecting funnel plots when less than ten datasets were available^26^, while Egger’s regression test was used when ten or more independent datasets were available^23^. We performed the Duval and Tweedie trim and fill test to adjust ESs when evidence of publication bias was found^27^. Sensitivity analysis where one study at a time was excluded from the analysis was performed to verify whether an outlier could be biasing our ES estimates^28^.

To evaluate potential sources of heterogeneity and confounding effects, we performed meta-regression and subgroup meta-analyses. Specifically, when there were at least five datasets, we conducted the meta-regression procedure using the unrestricted maximum likelihood method. Regarding subgroup meta-analysis, we focused on the studies that included different dosages of methylphenidate. Specifically, we subdivided the included studies according to the mean dosage of methylphenidate with the cut-off point set at 0.6 mg/day (*i.e*., the low-dose subgroup ≤ 0.6 mg/day and the high-dose subgroup > 0.6 mg/day). This cut-off point was previously used in a meta-analysis in Cochrane Systematic Reviews^29^. We performed subgroup analysis when data from at least three independent studies were provided^29^. In addition, we used interaction tests to evaluate differences in ESs between the subgroups^30^. Statistical significance was set at a two-tailed *alpha* level of 0.05.

## Results

### Study selection

**Figure 1:**
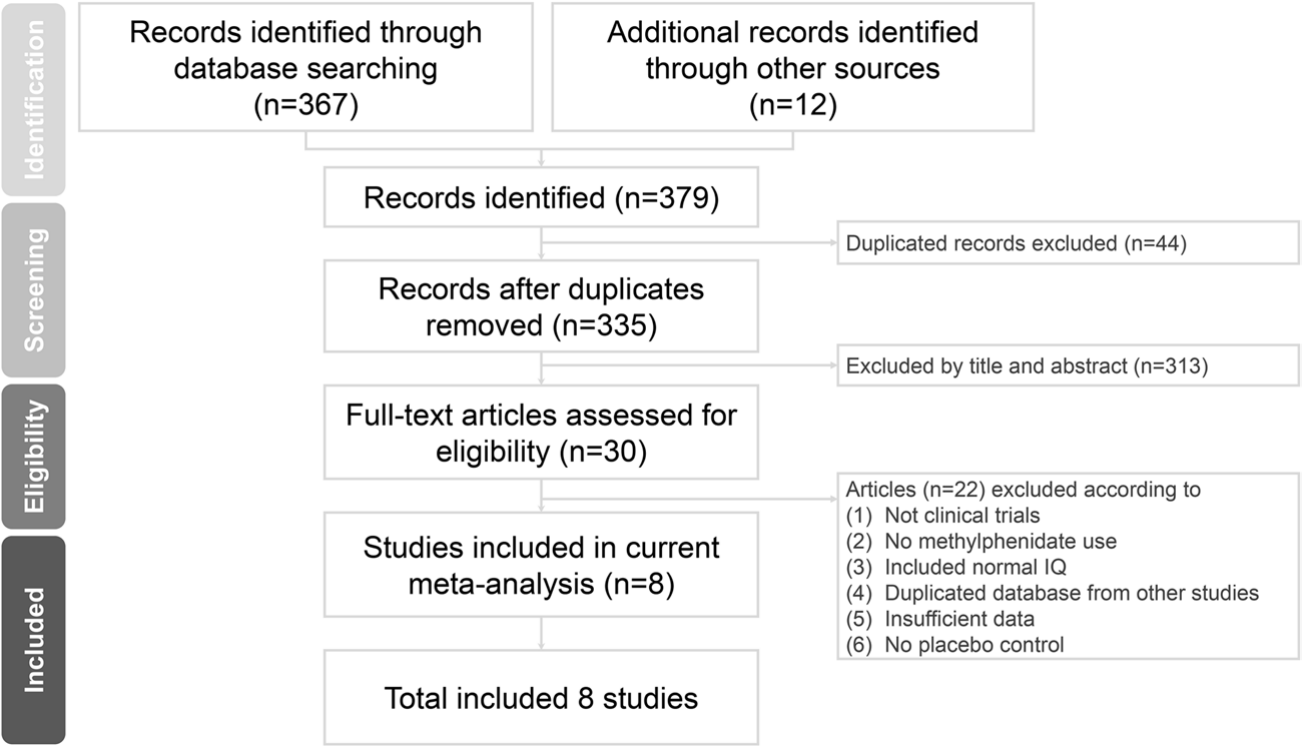
Flowchart of literature retrieval and appraisal for the current systematic review and meta-analysis.

The PRISMA flowchart used to select the studies in this systematic review is shown in Fig. 1. After excluding duplicates, 30 full-text articles were assessed for eligibility. Among them, 22 were excluded (see Supplementary Table 2 for the reasons why these studies were excluded). Therefore, eight articles were eligible for the current meta-analysis (Table 1)^4,13–18,31^.

**Table 1:** Summary of characteristics of studies in the current meta-analysis.

Study (year)	Diagnosis	Criteria	Design	Comparison	N	Duration (weeks)	Outcome	Mean age (years)	Female	Country
Simonoff E (2013)	1. Hyperkinetic disorder 2. IQ = 30–69	ICD 10	RCT	1. MPH-IR 0.5–1.5 mg/kg/day (adjusted by clinical response)	61	16	Teacher: Conner ADHD index (+) Connerhyperactivity (+) ABC hyperactivity (+) Parents: Conner ADHD index (+) Conner hyperactivity (+) ABC hyperactivity (+)	10.83	26%	UK
				2. Placebo	61			11.5	34%	
Handen BL (1999)	1. ADHD 2. IQ = 40–78	DSM-III-R	Double-blind Crossover	1. MPH 0.6 mg/kg/day	11	1	Teacher: Conner Hyperactivity index (+) Conner Hyperactivity subscale (+) Conner Conduct subscale (−) Conner Inattention subscale (+)	4.9	18%	USA
				2. MPH 1.2 mg/kg/day	11					
				3. Placebo	11					
Aman MG (1997)	1. ADHD 2. IQ < 85	DSM-III-R	Double-blind Crossover	1. MPH 0.4 mg/kg/day	30	2	Teacher: Conner Abbreviated (-) Conner Hyperactivity subscale (+) Conner Conduct subscale Conner Inattention subscale ABC hyperactivity (+) Parents: Conner Abbreviated (-) ABC hyperactivity (-)	7.7	26.7%	USA
				2. Fenfluramine 1–2 mg/kg/day	30					
				3. Placebo	30					
Aman MG (1993)	1. ADHD 2. IQ ≦ 78	DSM-III-R	Double-blind Crossover	1. MPH 0.4 mg/kg/day	28	4	Teacher: Conner Hyperactivity subscale (+) Conner Conduct subscale (+) Conner Inattention subscale (+) ABC hyperactivity (+) Parents: Conner Abbreviated (-) ABC hyperactivity (+)	8.8	28.6%	USA
				2. Fenfluramine 1.5 mg/kg/day	28					
				3. Placebo	28					
Handen BL (1992)	1. ADHD 2. IQ < 80	DSM-III-R	Double-blind Crossover	1. MPH 0.6 mg/kg/day	14	3	Teacher: Conner Hyperactivity index (+) Conner Hyperactivity subscale (+) Conner Conduct subscale (−) Conner Inattention subscale (+)	9.1	28.6%	USA
				2. MPH 1.2 mg/kg/day	14					
				3. Placebo	14					
Handen BL (1990)	1. ADHD 2. IQ < 80	DSM-III-R	Double-blind Crossover	1. MPH 0.6 mg/kg/day	12	3	Teacher: Conner Hyperactivity index (+) Conner Hyperactivity subscale (+) Conner Conduct subscale (+) Conner Inattention subscale (−/+)	6–9	8.3%	USA
				2. MPH 1.2 mg/kg/day	12					
				3. Placebo	12					
Hagerman RJ (1988)	1. Attention problems 2. Fragile X syndrome (IQ ≦ 77)	Conner’s rating scale	Double-blind Crossover	1. MPH 0.6 mg/kg/day	15	1	Teacher: Conner Abbreviated (−) Parents: Conner Abbreviated (-)	7.9	13%	USA
				2. Dextroamphetamin 0.2 mg/kg/day	15					
				3. Placebo	15					
Varley CK (1982)	1. Attention problems 2. IQ ≦ 77	DSM-III	Double-blind Crossover	1. MPH 0.3 mg/kg/day	10	1	Teacher: Conner Hyperactivity index (+/−) Parents: Conner Hyperactivity index (+/−)	11.33	30%	USA
				2. MPH 0.6 mg/kg/day	10					
				3. Placebo	10					

Among the eight eligible articles, 242 subjects received methylphenidate (mean age = 9.17 years, mean female proportion = 26.9%, median treatment duration = 2.5 weeks) and 181 subjects receiving placebo (mean age = 9.42 years, mean female proportion = 26.9%). Of the eight studies, five recruited subjects diagnosed according to the DSM-III-R^13–15,18,31^, one according to the ICD-10^4^, one according to the DSM-III^17^, and one according to the Conners’ Parent or Teacher Rating Scale^16^.

With regards to ADHD symptom evaluation, all eight articles^4,13–18,31^ evaluated symptoms using the Conners’ Teacher Rating Scale (CTRS)^32^, three^4,17,31^ with the Aberrant Behavior Checklist (ABC)^33^, and only two^4,31^ with Conners’ Parents Rating Scale (CPRS)^34^. Three articles also evaluated changes in CPT scores^14,16,18^.

### Methodological quality of the included studies

Among the eight studies, the average Jadad score was 2.5 with a standard deviation (SD) of 1.07 (Supplementary Table 3).

### Primary outcome: treatment effect on ADHD symptoms as assessed by changes in severity

#### Primary outcome: overall severity

**Figure 2:**
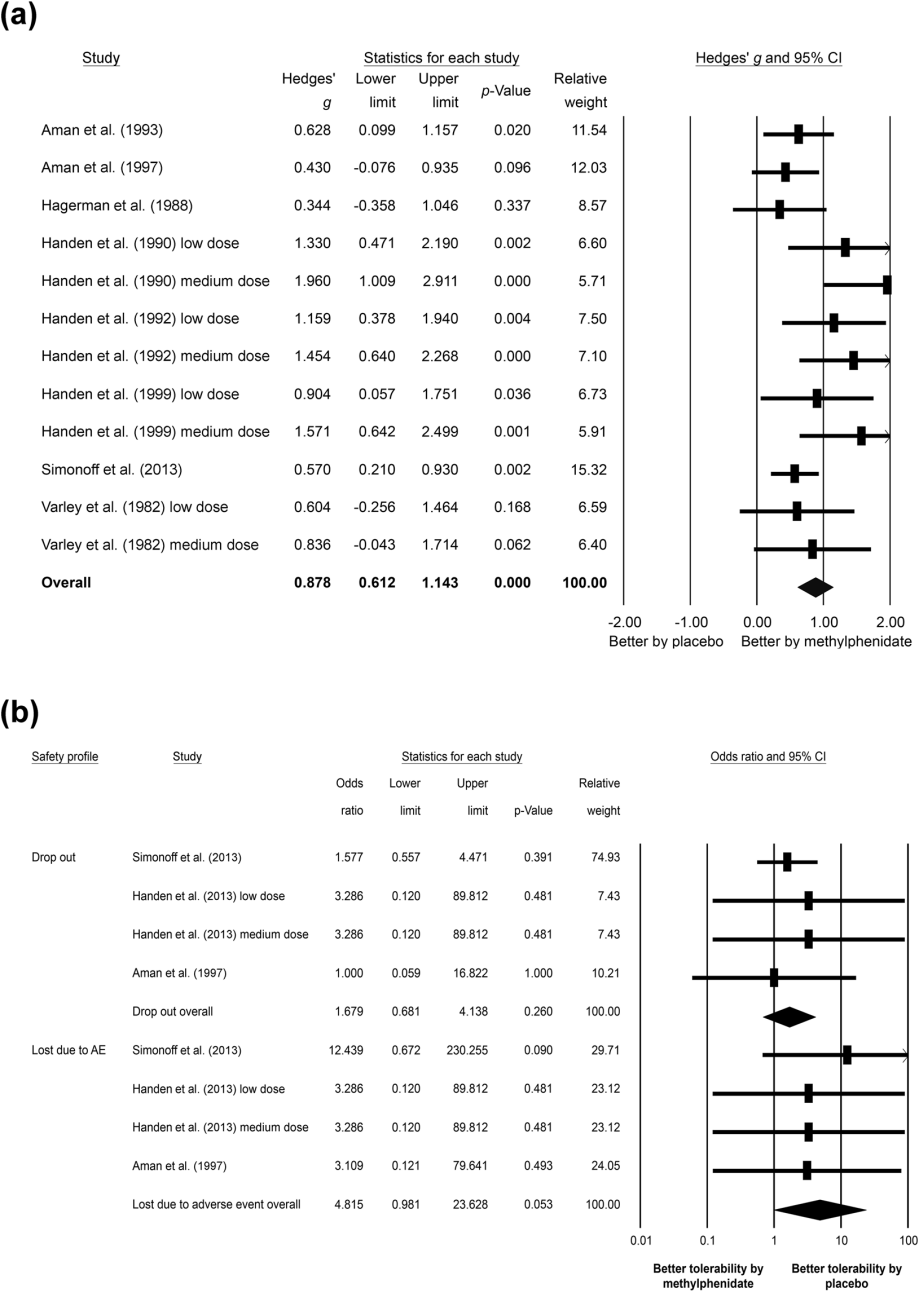
Forest plot of (**a**) primary outcome of the current meta-analysis, showing significant improvements in the overall severity of attention-deficit/hyperactivity disorder (ADHD) after methylphenidate treatment compared to that in placebo groups (Hedges’ *g* = 0.878, 95% CI = 0.612 to 1.143, *p* < 0.001), and (**b**) secondary outcome of the current meta-analysis, indicating insignificant difference in safety profile between methylphenidate and placebo treatments as assessed by drop-out rate (odds ratio = 1.679, 95% CI = 0.681 to 4.138, *p* = 0.260) and rate of treatment discontinuation due to adverse events (odds ratio = 4.815, 95% CI = 0.981 to 23.628, *p* = 0.053).

The meta-analysis of the eight studies with twelve datasets evaluating the changes in overall ADHD severity^4,13–18,31^, showed that the subjects receiving methylphenidate had better improvements in overall ADHD severity than those receiving placebo (*k* = 12, Hedges’ *g* = 0.878, 95% CI = 0.612 to 1.143, *p* < 0.001) (Fig. 2(a)) without significant heterogeneity (Q value = 19.027, df = 11, *I*^2^ = 42.188%, *p* = 0.061) but with significant publication bias via Egger’s regression test (t = 3.093, df = 10, *p* = 0.011). The adjusted ESs by Duval and Tweedie’s trim and fill test remained statistically significant (adjusted Hedges’ *g* = 0.641, 95% CI = 0.338 to 0.943).

#### Sensitivity analysis

The sensitivity test where one study at a time was excluded from the analysis revealed that the significant results of the meta-analysis did not change after the removal of any one of the included studies. Therefore, the significance of the results was not due to any outliers among the included studies.

#### Meta-regression

Meta-regression suggested a significant positive association between changes in overall ADHD severity and methylphenidate dosage (*k* = 11, slope = 1.334, *p* < 0.001) with no significant associations between changes in overall ADHD severity and mean age (*p* = 0.366), female proportion (*p* = 0.112), mean IQ (*p* = 0.119), or treatment duration (*p* = 0.465).

### Secondary outcomes: treatment effect on ADHD symptoms as assessed by changes in subscales of different dimensions of CTRS

#### Secondary outcome: conduct problem severity as assessed by CTRS-conduct

**Figure 3:**
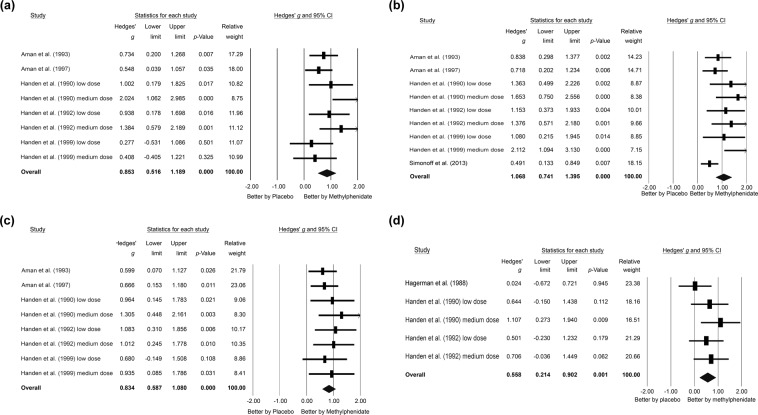
Forest plot of secondary outcomes of the current meta-analysis, demonstrating significant improvements in (**a**) CTRS-conduct subscales (Hedges’ *g* = 0.853, 95% CI = 0.516 to 1.189, *p* < 0.001), (**b**) CTRS-hyperactive subscales (Hedges’ *g* = 1.068, 95% CI = 0.741 to 1.395, *p* < 0.001), (**c**) CTRS-inattention subscales (Hedges’ *g* = 0.834, 95% CI = 0.587 to 1.080, *p* < 0.001), and (**d**) CPT scores (Hedges’ *g* = 0.558, 95% CI = 0.214 to 0.902, *p* = 0.001) after methylphenidate treatment compared to the placebo groups. Abbreviations: ADHD: Attention deficit hyperactivity disorder; AE: adverse event; CI: confidence interval; CPT: continuous performance test; CTRS: Conners’ Teacher Rating Scale; MA: meta-analysis.

**Figure 4:**
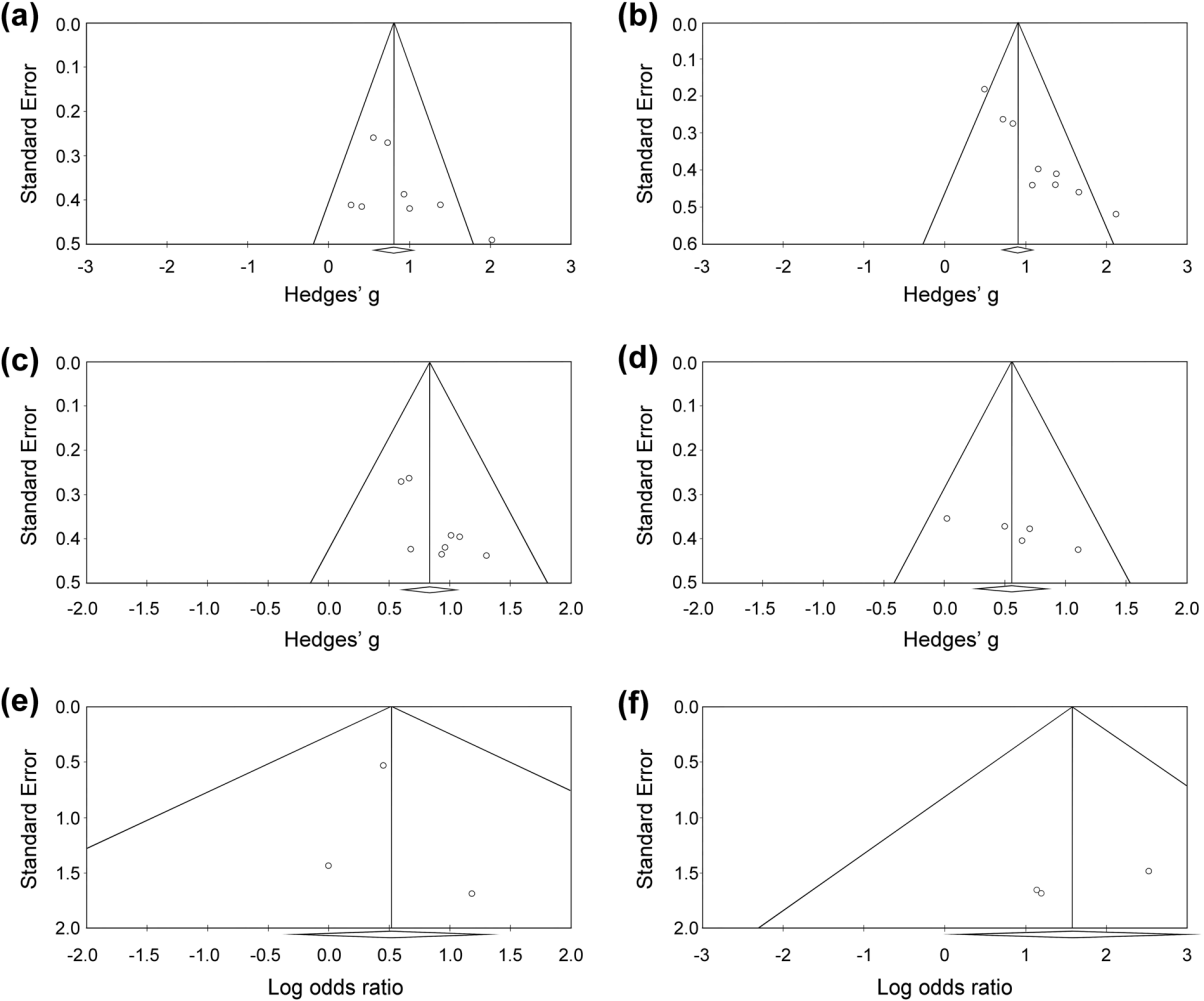
Funnel plots of standard error for (**a**) Conners’ Teacher Rating Scale (CTRS)-conduct, (**b**) CTRS-hyperactivity, (**c**) CTRS-inattention, and (**d**) continuous performance test (CPT) by Hedges’ g; (**e**) drop-out rate, and (**f**) rate of treatment discontinuation due to adverse events by log odds ratio.

When focusing on the five studies^14,15,17,18,31^ with eight datasets evaluating changes in CTRS-conduct, the meta-analysis showed that the subjects receiving methylphenidate had better improvements in CTRS-conduct severity than those receiving placebo (*k* = 8, Hedges’ *g* = 0.853, 95% CI = 0.516 to 1.189, *p* < 0.001; MDs = 0.816) (Fig. 3(a)) without significant heterogeneity (Q value = 12.089, df = 7, *I*^2^ = 42.098%, *p* = 0.098) or publication bias via inspection of the funnel plot (Fig. 4(a)).

#### Secondary outcome: hyperactive severity as assessed by CTRS-hyperactive

An investigation into the six studies^4,14,15,17,18,31^ with nine datasets evaluating changes in CTRS-hyperactive demonstrated that the subjects receiving methylphenidate had better improvements in CTRS-hyperactive severity compared to those receiving placebo (*k* = 9, Hedges’ *g* = 1.068, 95% CI = 0.741 to 1.395, *p* < 0.001; MDs = 1.976) (Fig. 3(b)) with significant heterogeneity (Q value = 16.687, df = 8, *I*^2^ = 52.058%, *p* = 0.034) and significant publication bias via inspection of the funnel plot (Fig. 4(b)). The adjusted ESs by Duval and Tweedie’s trim and fill test remained statistically significant (adjusted Hedges’ *g* = 0.694, 95% CI = 0.348 to 1.041).

#### Secondary outcome: inattention severity as assessed by CTRS-inattention

Considering the five studies^14,15,17,18,31^ with eight datasets evaluating changes in CTRS-inattention, the meta-analysis showed that the subjects receiving methylphenidate had better improvements in CTRS-inattention severity than those receiving placebo (*k* = 8, Hedges’ *g* = 0.834, 95% CI = 0.587 to 1.080, *p* < 0.001; MDs = 0.742) (Fig. 3(c)) without significant heterogeneity (Q value = 3.221, df = 7, *I*^2^ < 0.001%, *p* = 0.864) but significant publication bias via inspection of the funnel plot (Fig. 4(c)). The adjusted ESs by Duval and Tweedie’s trim and fill test remained statistically significant (adjusted Hedges’ *g* = 0.717, 95% CI = 0.500 to 0.934).

#### Subgroup meta-analysis: treatment effect on ADHD symptoms with different methylphenidate dosages

Subgroup meta-analysis showed that the subjects receiving methylphenidate had better improvements in overall ADHD severity than those receiving placebo both in the low-dose (*k* = 8, Hedges’ *g* = 0.694, 95% CI = 0.448 to 0.940, *p* < 0.001) and high-dose (*k* = 3, Hedges’ *g* = 1.638, 95% CI = 1.123 to 2.153, *p* < 0.001) subgroups^13–18,31^. Furthermore, the ESs were significantly higher in the high-dose subgroup than those in the low-dose subgroup by the interaction test (*p* = 0.001)^14,15,18^.

#### Secondary outcome: treatment effect on continuous performance as assessed by changes in CPT

When focusing on the three studies^14,16,18^ that investigated differences in CPT results between placebo and methylphenidate treatment groups, the meta-analysis showed that the subjects receiving methylphenidate had better improvements in CPT results than those receiving placebo (*k* = 5, Hedges’ *g* = 0.558, 95% CI = 0.214 to 0.902, *p* = 0.001; MDs = 11.955) (Fig. 3(d)) without significant heterogeneity (Q value = 4.141, df = 4, *I*^2^ = 3.405%, *p* = 0.387) but significant publication bias via inspection of the funnel plot (Fig. 4(d)). The adjusted ESs by Duval and Tweedie’s trim and fill test remained statistically significant (adjusted Hedges’ *g* = 0.368, 95% CI = 0.009 to 0.727).

#### Secondary outcome: tolerability assessed by drop-out rate and rate of treatment discontinuation due to adverse events

The current meta-analysis showed no significant difference in drop-out rate between subjects receiving methylphenidate and those taking placebos (*k* = 4, OR = 1.679, 95% CI = 0.681 to 4.138, *p* = 0.260) (Fig. 2(b)). Forest plot demonstrated no significant heterogeneity (Q value = 0.460, df = 3, *I*^2^ < 0.001%, *p* = 0.928) or significant publication bias (Fig. 4(e)).

Similarly, there was no significant difference in the rate of treatment discontinuation due to adverse events between subjects treated with methylphenidate and those receiving placebos (*k* = 4, OR = 4.815, 95% CI = 0.981 to 23.628, *p* = 0.053) (Fig. 2(b)). Despite the lack of significant heterogeneity (Q value = 0.579, df = 3, *I*^2^ < 0.001%, *p* = 0.901), significant publication bias was noted via inspection of the funnel plot (Fig. 4(f)). The adjusted ESs by Duval and Tweedie’s trim and fill test showed significantly higher rate of treatment discontinuation due to adverse events in subjects receiving methylphenidate than those taking placebos (adjusted OR = 5.531, 95% CI = 1.326 to 23.069).

## Discussion

To the best of our knowledge, this is the first meta-analysis to address the treatment effect of methylphenidate on children with ADHD and ID or borderline intellectual functioning. Our meta-analysis suggested that children with borderline intellectual functioning or ID receiving methylphenidate experienced better improvements than those receiving placebo in both overall ADHD severity (*p* < 0.001) and ADHD-specific symptoms, including CTRS-conduct (*p* < 0.001), CTRS-hyperactivity (*p* < 0.001), CTRS-inattention (*p* < 0.001) and CPT results (*p* = 0.001). Furthermore, our meta-regression analysis also showed that the efficacy of methylphenidate was related to its dosage. Finally, there was no significant difference in drop-out rate or rate of treatment discontinuation due to adverse events between subjects receiving methylphenidate and those taking placebos.

The main findings of the current meta-analysis were the beneficial effects of methylphenidate on the symptoms of ADHD, both in primary outcome (overall disease severity) and secondary outcomes (hyperactivity, conduct, inattention and CPT results) in children with ADHD and ID or borderline intellectual functioning. These findings are similar to those in previous meta-analyses conducted in children with ADHD and a normal IQ^10,35^. Therefore, our findings further support the potentially beneficial effect of methylphenidate against ADHD symptoms, which has not been well studied previously and has shown controversial results in this study population^14,15,17,18^.

Our study demonstrated that the efficacy of methylphenidate treatment increased with an increased dosage, as shown in our meta-regression analysis (slope = 1.334, *p* < 0.001) and subgroup analyses (Hedges’ *g* = 0.694 in the low-dose subgroup, Hedges’ *g* = 1.638 in the high-dose subgroup, interaction test between the two subgroups: *p* = 0.001). These findings may prompt the development of new strategies in clinical practice. Currently, the most common strategy for children taking methylphenidate in clinical practice is “dose optimization”, which is defined as “start with a low dose and titrate gradually upward to an optimal low-dose”^36^. The different treatment effect of methylphenidate found in the current meta-analysis may be partially supported by a previous meta-analysis conducted in children with ADHD and a normal IQ^10^. In that meta-analysis, although the overall results of the meta-analysis were based on both randomized clinical trials and cross-over trials and did not find any difference in treatment efficacy between low-dose (≦0.6 mg per day) or high-dose (>0.6 mg per day) treatment, the treatment effects became significantly higher in the high-dose group if they only focused on trials with a cross-over design^10^. This interesting finding may provide some evidence for individual variations in dose response. Moreover, it has been shown that certain individuals may respond to a higher dose of methylphenidate, but that substantial inter-subject variability may exist in response to methylphenidate dosage^37^. However, given the potentially higher incidence of adverse events in patients with subaverage IQ^4^, further large-scale randomized controlled trials about the potential adverse events in children with ADHD and ID or borderline intellectual function are warranted.

Finally, with regards to the safety and tolerability of methylphenidate in children with ADHD and ID or borderline intellectual functioning, we did not find significant difference in drop-out rate (OR = 1.679, *p* = 0.260) or rate of treatment discontinuation due to adverse events (OR = 4.815, *p* = 0.053) between subjects treated with methylphenidate and those receiving placebos. Moreover, most studies reported that methylphenidate was well tolerated without reports of severe adverse events (Supplementary Table 4). Overall, methylphenidate seemed well tolerated in the studied populations.

### Limitations

There are several limitations to the present meta-analysis that should be addressed prior to its application in clinical practice. First, the heterogeneous nature of the included studies should be noted, including the wide range of dosages of methylphenidate (0.6 to 1.2 mg/kg/day), wide range of methylphenidate treatment duration (1–16 weeks), short treatment duration in most of the included studies (seven of the eight studies had a treatment duration of between 1 and 4 weeks), male predominance (73.1%), and mainly Caucasian ethnicity. In addition, most of the included studies were cross-over studies with quality being ranked as poor in five of the eight studies. Second, the number of the included studies and the overall sample sizes were relatively small. Third, only one of the included studies was published in the last decade.

## Conclusions

The current meta-analysis provides evidence in support of a significant dose-dependent beneficial effect of methylphenidate on overall ADHD severity, conduct, hyperactivity, and inattentive symptoms in children with ADHD and ID or borderline intellectual functioning. Further large-scale well-designed randomized clinical trials investigating the treatment efficacy and tolerability of methylphenidate in this population are warranted.

## Supplementary information

Supplementary Tables

## References

[1] American Psychiatric Association. Attention-deficit/ hyperactivity disorder. Diagnostic and Statistical Manual of Mental Disorders. 5th edition. Washington, DC., 59–65 (2013).

[2] Brown KA, Samuel S, Patel DR (2018). Pharmacologic management of attention deficit hyperactivity disorder in children and adolescents: a review for practitioners. Transl. Pediatr..

[3] Simonoff E, Pickles A, Wood N, Gringras P, Chadwick O (2007). ADHD symptoms in children with mild intellectual disability. J. Am. Acad. Child Adolesc. Psychiatry..

[4] Simonoff E (2013). Randomized controlled double-blind trial of optimal dose methylphenidate in children and adolescents with severe attention deficit hyperactivity disorder and intellectual disability. J Child Psychol Psychiatry.

[5] Charach, A. *et al*. Attention Deficit Hyperactivity Disorder: Effectiveness of Treatment in At-Risk Preschoolers; Long-Term Effectiveness in All Ages; and Variability in Prevalence, *Diagnosis, and Treatment [Internet]* (2011). 22191110

[6] Pliszka SR (2006). The Texas Children’s Medication Algorithm Project: revision of the algorithm for pharmacotherapy of attention-deficit/hyperactivity disorder. J Am Acad Child Adolesc Psychiatry.

[7] Briars L, Todd T (2016). A Review of Pharmacological Management of Attention-Deficit/Hyperactivity Disorder. J Pediatr Pharmacol Ther.

[8] MTA Cooperative Group (1999). A 14-month randomized clinical trial of treatment strategies for attention-deficit/hyperactivity disorder. The MTA Cooperative Group. Multimodal Treatment Study of Children with ADHD. Arch Gen psychiatry.

[9] Chan E, Fogler JM, Hammerness PG (2016). Treatment of Attention-Deficit/Hyperactivity Disorder in Adolescents: A Systematic Review. Jama.

[10] Storebo, O. J. *et al*. Methylphenidate for children and adolescents with attention deficit hyperactivity disorder (ADHD). *Cochrane Database Syst Rev*, CD009885, 10.1002/14651858.CD009885.pub2 (2015). 10.1002/14651858.CD009885.pub2 PMC8763351 26599576

[11] Padilha S, Virtuoso S, Tonin FS, Borba HHL, Pontarolo R (2018). Efficacy and safety of drugs for attention deficit hyperactivity disorder in children and adolescents: a network meta-analysis. Eur Child Adolesc Psychiatry.

[12] Aman MG, Buican B, Arnold LE (2003). Methylphenidate treatment in children with borderline IQ and mental retardation: analysis of three aggregated studies. Journal of child and adolescent psychopharmacology.

[13] Varley CK, Trupin EW (1982). Double-blind administration of methylphenidate to mentally retarded children with attention deficit disorder; a preliminary study. Am J Ment Defic.

[14] Handen BL (1992). Effects and noneffects of methylphenidate in children with mental retardation and ADHD. J Am Acad Child Adolesc Psychiatry.

[15] Handen BL, Feldman HM, Lurier A, Murray PJ (1999). Efficacy of methylphenidate among preschool children with developmental disabilities and ADHD. J Am Acad Child Adolesc Psychiatry.

[16] Hagerman RJ, Murphy MA, Wittenberger MD (1988). A controlled trial of stimulant medication in children with the fragile X syndrome. Am J Med Genet.

[17] Aman MG, Kern RA, McGhee DE, Arnold LE (1993). Fenfluramine and methylphenidate in children with mental retardation and ADHD: clinical and side effects. J Am Acad Child Adolesc Psychiatry.

[18] Handen BL, Breaux AM, Gosling A, Ploof DL, Feldman H (1990). Efficacy of methylphenidate among mentally retarded children with attention deficit hyperactivity disorder. Pediatrics.

[19] Tarrant N (2018). The effectiveness of methylphenidate in the management of Attention Deficit Hyperactivity Disorder (ADHD) in people with intellectual disabilities: A systematic review. Res Dev Disabil..

[20] Liberati A (2009). The PRISMA statement for reporting systematic reviews and meta-analyses of studies that evaluate health care interventions: explanation and elaboration. PLoS Med..

[21] Sturman N, Deckx L, van Driel ML (2017). Methylphenidate for children and adolescents with autism spectrum disorder. The Cochrane database of systematic reviews.

[22] Jadad AR (1996). Assessing the quality of reports of randomized clinical trials: is blinding necessary?. Controlled clinical trials.

[23] Egger M, Davey Smith G, Schneider M, Minder C (1997). Bias in meta-analysis detected by a simple, graphical test. Bmj.

[24] Higgins JP, Thompson SG (2002). Quantifying heterogeneity in a meta-analysis. Stat Med.

[25] Borenstein M, Higgins JP, Hedges LV, Rothstein HR (2017). Basics of meta-analysis: I(2) is not an absolute measure of heterogeneity. Res Synth Methods.

[26] Higgins, J. P. & Green, S. In *Cochrane Handbook for Systematic Reviews of Interventions* (eds Higgins, J. P. & Green, S.) (Cochrane Library, 2011).

[27] Duval S, Tweedie R (2000). Trim and fill: A simple funnel-plot-based method of testing and adjusting for publication bias in meta-analysis. Biometrics.

[28] Tobias A (1999). Assessing the influence of a single study in meta-analysis. Stata Tech Bull.

[29] Davey J, Turner RM, Clarke MJ, Higgins JP (2011). Characteristics of meta-analyses and their component studies in the Cochrane Database of Systematic Reviews: a cross-sectional, descriptive analysis. BMC medical research methodology.

[30] Altman DG, Bland JM (2003). Interaction revisited: the difference between two estimates. BMJ.

[31] Aman MG (1997). Fenfluramine and methylphenidate in children with mental retardation and borderline IQ: clinical effects. Am J Ment Retard.

[32] Conners CK (1969). A teacher rating scale for use in drug studies with children. The American journal of psychiatry.

[33] Aman, M. & Singh, N. *The Aberrant Behavior Checklist-Community*. (Slosson Education Publications, Inc., 1994).

[34] Conners CK, Sitarenios G, Parker JD, Epstein JN (1998). The revised Conners’ Parent Rating Scale (CPRS-R): factor structure, reliability, and criterion validity. J Abnorm Child Psychol.

[35] Coghill DR (2014). Effects of methylphenidate on cognitive functions in children and adolescents with attention-deficit/hyperactivity disorder: evidence from a systematic review and a meta-analysis. Biological psychiatry.

[36] Dulcan MK (1990). Using psychostimulants to treat behavioral disorders of children and adolescents. Journal of child and adolescent psychopharmacology.

[37] Huss M (2017). Methylphenidate dose optimization for ADHD treatment: review of safety, efficacy, and clinical necessity. Neuropsychiatr Dis Treat.

