# Supplementary material for: Therapeutic effects of methylphenidate for attention-deficit/hyperactivity disorder in children with borderline intellectual functioning or intellectual disability: A systematic review and meta-analysis
Source: Sci Rep. 2019 Nov 4;9:15908. doi: 10.1038/s41598-019-52205-6 (PMC6828952; doi:10.1038/s41598-019-52205-6)
Supplement: Supplementary file 1 — Supplementary Tables [file 41598_2019_52205_MOESM1_ESM.docx]

**Therapeutic effects of methylphenidate for attention-deficit/hyperactivity disorder in children with borderline intellectual functioning or intellectual disability: A systematic review and meta-analysis**

*Running title: Methylphenidate in ADHD with MR*

Cheuk-Kwan Sun^1,†^, Ping-Tao Tseng^2,†^, Ching-Kuan Wu^3^, Dian-Jeng Li^4,5^, Tien-Yu Chen^6,7^, Brendon Stubbs^8,9,10^, Andre F Carvalho^11,12^, Yen-Wen Chen^13^, Pao-Yen Lin^14,15^ , Yu-Shian Cheng^3,^* & Ming-Kung Wu^14,^*

^1^ Department of Emergency Medicine, E-Da Hospital, I-Shou University School of Medicine for International Students, Kaohsiung, Taiwan

^2^ WinShine Clinics in Specialty of Psychiatry, Kaohsiung, Taiwan

^3^ Department of Psychiatry, Tsyr-Huey Mental Hospital, Kaohsiung Jen-Ai’s Home, Kaohsiung, Taiwan

^4^ Graduate Institute of Medicine, College of Medicine, Kaohsiung Medical University, Kaohsiung, Taiwan

^5^ Department of Addiction Science, Kaohsiung Municipal Kai-Syuan Psychiatric Hospital, Kaohsiung, Taiwan

^6^ Department of Psychiatry, Tri-Service General Hospital; School of Medicine, National Defense Medical Center, Taipei, Taiwan

^7^ Institute of Brain Science, National Yang-Ming University, Taipei, Taiwan

^8^ Physiotherapy Department, South London and Maudsley NHS Foundation Trust, London, UK

^9^ Department of Psychological Medicine, Institute of Psychiatry, Psychology and Neuroscience (IoPPN), King's College London, De Crespigny Park, London, UK

^10^ Faculty of Health, Social Care and Education, Anglia Ruskin University, Chelmsford, UK

^11^ Department of Psychiatry, University of Toronto, Toronto, ON, Canada.

^12^ Centre for Addiction & Mental Health (CAMH), Toronto, ON, Canada

^13^ Prospect Clinic for Otorhinolaryngology & Neurology

^14^ Department of Psychiatry, Kaohsiung Chang Gung Memorial Hospital and Chang Gung University College of Medicine, Kaohsiung, Taiwan

^15^ Institute for Translational Research in Biomedical Sciences, Kaohsiung Chang Gung Memorial Hospital, Kaohsiung, Taiwan

^†^ Contributed equally as the first author

* Contributed equally as the corresponding author

**Please address correspondence to:**

Yu-Shian Cheng, MD

Affiliation: Department of Psychiatry, Tsyr-Huey Mental Hospital, Kaohsiung Jen-Ai’s Home, Taiwan

Address: No.509, Fengping 1^st^ Rd., Daliao Dist., Kaohsiung City 831, Taiwan

Telephone: 886-7-7030315

Fax: 886-7-7012624

Email: n043283@gmail.com

OR

Ming-Kung Wu, MD

Affiliation: Department of Psychiatry, Kaohsiung Chang Gung Memorial Hospital and Chang Gung University College of Medicine, Kaohsiung, Taiwan

Address: No.123, Dapi Rd., Niaosong Dist., Kaohsiung City 833, Taiwan

Telephone: 886-7-7317123

Email: [mingkung180@gmail.com](mailto:mingkung180@gmail.com)

**Supplementary Table 1.** PRISMA checklist of current meta-analysis

| **Section/Topic** | **#** | **Checklist Item** | **Reported on Page #** |
| --- | --- | --- | --- |
| **TITLE** | | | |
| Title | 1 | Identify the report as a systematic review, meta-analysis, or both. | 1 |
| **ABSTRACT** | | | |
| Structured summary | 2 | Provide a structured summary including, as applicable: background; objectives; data sources; study eligibility criteria, participants, and interventions; study appraisal and synthesis methods; results; limitations; conclusions and implications of key findings; systematic review registration number. | 4 |
| **INTRODUCTION** | | | |
| Rationale | 3 | Describe the rationale for the review in the context of what is already known. | 5-6 |
| Objectives | 4 | Provide an explicit statement of questions being addressed with reference to participants, interventions, comparisons, outcomes, and study design (PICOS). | 5-6 |
| **METHODS** | | | |
| Protocol and registration | 5 | Indicate if a review protocol exists, if and where it can be accessed (e.g., Web address), and, if available, provide registration information including registration number. | 7-8 |
| Eligibility criteria | 6 | Specify study characteristics (e.g., PICOS, length of follow-up) and report characteristics (e.g., years considered, language, publication status) used as criteria for eligibility, giving rationale. | 7-8 |
| Information sources | 7 | Describe all information sources (e.g., databases with dates of coverage, contact with study authors to identify additional studies) in the search and date last searched. | 7-8 |
| Search | 8 | Present full electronic search strategy for at least one database, including any limits used, such that it could be repeated. | 8-9 |
| Study selection | 9 | State the process for selecting studies (i.e., screening, eligibility, included in systematic review, and, if applicable, included in the meta-analysis). | 8-9 |
| Data collection process | 10 | Describe method of data extraction from reports (e.g., piloted forms, independently, in duplicate) and any processes for obtaining and confirming data from investigators. | 8-9 |
| Data items | 11 | List and define all variables for which data were sought (e.g., PICOS, funding sources) and any assumptions and simplifications made. | 8-9 |
| Risk of bias in individual studies | 12 | Describe methods used for assessing risk of bias of individual studies (including specification of whether this was done at the study or outcome level), and how this information is to be used in any data synthesis. | 8-9 |
| Summary measures | 13 | State the principal summary measures (e.g., risk ratio, difference in means). | 8-9 |
| Synthesis of results | 14 | Describe the methods of handling data and combining results of studies, if done, including measures of consistency (e.g., I^2^) for each meta-analysis. | 9-10 |
| Risk of bias across studies | 15 | Specify any assessment of risk of bias that may affect the cumulative evidence (e.g., publication bias, selective reporting within studies). | 9-10 |
| Additional analyses | 16 | Describe methods of additional analyses (e.g., sensitivity or subgroup analyses, meta-regression), if done, indicating which were pre-specified. | 9-10 |
| **RESULTS** | | | |
| Study selection | 17 | Give numbers of studies screened, assessed for eligibility, and included in the review, with reasons for exclusions at each stage, ideally with a flow diagram. | 11-13 |
| Study characteristics | 18 | For each study, present characteristics for which data were extracted (e.g., study size, PICOS, follow-up period) and provide the citations. | 11-13, table 1 |
| Risk of bias within studies | 19 | Present data on risk of bias of each study and, if available, any outcome level assessment (see item 12). | 11-14 |
| Results of individual studies | 20 | For all outcomes considered (benefits or harms), present, for each study: (a) simple summary data for each intervention group (b) effect estimates and confidence intervals, ideally with a forest plot. | 12-13, table 1 |
| Synthesis of results | 21 | Present the main results of the review. If meta-analyses done, include for each, confidence intervals and measures of consistency. | 13-15 figure 2 |
| Risk of bias across studies | 22 | Present results of any assessment of risk of bias across studies (see Item 15). | 14-15 |
| Additional analysis | 23 | Give results of additional analyses, if done (e.g., sensitivity or subgroup analyses, meta-regression [see Item 16]). | 13-15, figure 2 |
| **DISCUSSION** | | | |
| Summary of evidence | 24 | Summarize the main findings including the strength of evidence for each main outcome; consider their relevance to key groups (e.g., healthcare providers, users, and policy makers). | 16-18 |
| Limitations | 25 | Discuss limitations at study and outcome level (e.g., risk of bias), and at review-level (e.g., incomplete retrieval of identified research, reporting bias). | 18 |
| Conclusions | 26 | Provide a general interpretation of the results in the context of other evidence, and implications for future research. | 19 |
| **FUNDING** | | | |
| Funding | 27 | Describe sources of funding for the systematic review and other support (e.g., supply of data); role of funders for the systematic review. | 20 |

*From:* Moher D, Liberati A, Tetzlaff J, Altman DG, The PRISMA Group (2009). Preferred Reporting Items for Systematic Reviews and Meta-Analyses: The PRISMA Statement. PLoS Med 6(6): e1000097. doi:10.1371/journal.pmed1000097

**Supplementary Table 2.** Reasons for excluding articles

Not clinical trials (n=5)

Schmidt B, Anderson PJ, Doyle LW, et al. Survival without disability to age 5 years after neonatal caffeine therapy for apnea of prematurity. *Jama.* Jan 18 2012;307(3):275-282.

Hasler G, Luckenbaugh DA, Snow J, et al. Reward processing after catecholamine depletion in unmedicated, remitted subjects with major depressive disorder. *Biological psychiatry.* Aug 1 2009;66(3):201-205.

Handen BL, Janosky J, McAuliffe S. Long-term follow-up of children with mental retardation/borderline intellectual functioning and ADHD. *Journal of abnormal child psychology.* Aug 1997;25(4):287-295.

Power TJ, Blum NJ, Jones SM, Kaplan PE. Brief report: response to methylphenidate in two children with Williams syndrome. *Journal of autism and developmental disorders.* Feb 1997;27(1):79-87.

Aman MG, Pejeau C, Osborne P, Rojahn J, Handen B. Four-year follow-up of children with low intelligence and ADHD. *Research in developmental disabilities.* Nov-Dec 1996;17(6):417-432.

Not using methylphenidate (n=7)

Capone GT, Goyal P, Grados M, Smith B, Kammann H. Risperidone use in children with Down syndrome, severe intellectual disability, and comorbid autistic spectrum disorders: a naturalistic study. *Journal of developmental and behavioral pediatrics : JDBP.* Apr 2008;29(2):106-116.

Trzepacz PT, Spencer TJ, Zhang S, Bangs ME, Witte MM, Desaiah D. Effect of atomoxetine on Tanner stage sexual development in children and adolescents with attention deficit/hyperactivity disorder: 18-month results from a double-blind, placebo-controlled trial. *Current medical research and opinion.* 2011;27 Suppl 2:45-52.

Torrioli MG, Vernacotola S, Peruzzi L, et al. A double-blind, parallel, multicenter comparison of L-acetylcarnitine with placebo on the attention deficit hyperactivity disorder in fragile X syndrome boys. *American journal of medical genetics. Part A.* Apr 1 2008;146a(7):803-812.

Hutchison SL, Ghuman JK, Ghuman HS, Karpov I, Schuster JM. Efficacy of atomoxetine in the treatment of attention-deficit hyperactivity disorder in patients with common comorbidities in children, adolescents and adults: a review. *Therapeutic advances in psychopharmacology.* 2016;6(5):317-334. http://cochranelibrary-wiley.com/o/cochrane/clcentral/articles/420/CN-01208420/frame.html.

Aman MG, Smith T, Arnold LE, et al. A review of atomoxetine effects in young people with developmental disabilities. *Research in developmental disabilities.* 2014/06/01/ 2014;35(6):1412-1424.

Aman MG, Kern RA, Arnold LE, McGhee DE. Fenfluramine and mental retardation. *Journal of the American Academy of Child and Adolescent Psychiatry.* May 1991;30(3):507-508.

Aman MG, Marks RE, Turbott SH, Wilsher CP, Merry SN. Clinical effects of methylphenidate and thioridazine in intellectually subaverage children. *Journal of the American Academy of Child and Adolescent Psychiatry.* Mar 1991;30(2):246-256.

Not included due to inclusion of normal IQ participants (n=4)

Ghuman JK, Aman MG, Lecavalier L, et al. Randomized, placebo-controlled, crossover study of methylphenidate for attention-deficit/hyperactivity disorder symptoms in preschoolers with developmental disorders. *Journal of child and adolescent psychopharmacology.* Aug 2009;19(4):329-339.

Aman MG, Marks RE, Turbott SH, Wilsher CP, Merry SN. Methylphenidate and thioridazine in the treatment of intellectually subaverage children: effects on cognitive-motor performance. *Journal of the American Academy of Child and Adolescent Psychiatry.* Sep 1991;30(5):816-824.

Blacklidge VY, Ekblad RL. The effectiveness of methylphenidate hydrochloride (ritalin) on learning and behavior in public school educable mentally retarded children. *Pediatrics.* May 1971;47(5):923-926.

Alexandris A, Lundell FW. Effect of thioridazine, amphetamine and placebo on the hyperkinetic syndrome and cognitive area in mentally deficient children. *Canadian Medical Association journal.* Jan 13 1968;98(2):92-96.

No placebo-controlled trials (n=2)

Correia Filho AG, Bodanese R, Silva TL, Alvares JP, Aman M, Rohde LA. Comparison of risperidone and methylphenidate for reducing ADHD symptoms in children and adolescents with moderate mental retardation. *Journal of the American Academy of Child and Adolescent Psychiatry.* Aug 2005;44(8):748-755.

Nct. Amantadine Versus Ritalin in the Treatment of Attention Deficit Hyperactivity Disorder (ADHD). *Https://clinicaltrials.gov/show/nct01099059.* 2010. http://cochranelibrary-wiley.com/o/cochrane/clcentral/articles/905/CN-01528905/frame.html.

Insufficient data (n=3)

Pearson DA, Santos CW, Roache JD, et al. Treatment effects of methylphenidate on behavioral adjustment in children with mental retardation and ADHD. *Journal of the American Academy of Child and Adolescent Psychiatry.* Feb 2003;42(2):209-216.

Bawden HN, MacDonald GW, Shea S. Treatment of children with Williams syndrome with methylphenidate. *Journal of child neurology.* Jun 1997;12(4):248-252.

Handen BL, Janosky J, McAuliffe S, Breaux AM, Feldman H. Prediction of response to methylphenidate among children with ADHD and mental retardation. *Journal of the American Academy of Child and Adolescent Psychiatry.* Oct 1994;33(8):1185-1193.

Duplicated database from other studies (n=1)

Aman MG, Buican B, Arnold LE. Methylphenidate treatment in children with borderline IQ and mental retardation: analysis of three aggregated studies. *Journal of child and adolescent psychopharmacology.* Spring 2003;13(1):29-40.

**Supplementary Table 3.** Jadad scores of included studies in the current meta-analysis

| Study (year) | Randomization | Double blinding | Withdrawal and dropouts | Total scores | Quality appraisal |
| --- | --- | --- | --- | --- | --- |
| Simonoff E (2013) | 2 | 1 | 1 | 4 | Good |
|  |  |  |  |  |  |
| Handen BL (1999) | 1 | 2 | 1 | 4 | Good |
|  |  |  |  |  |  |
|  |  |  |  |  |  |
| Aman MG (1997) | 1 | 1 | 1 | 3 | Good |
|  |  |  |  |  |  |
|  |  |  |  |  |  |
| Aman MG (1993) | 1 | 1 | 0 | 2 | Poor |
|  |  |  |  |  |  |
|  |  |  |  |  |  |
| Handen BL (1992) | 0 | 1 | 0 | 1 | Poor |
|  |  |  |  |  |  |
|  |  |  |  |  |  |
| Handen BL (1990) | 0 | 1 | 1 | 2 | Poor |
|  |  |  |  |  |  |
|  |  |  |  |  |  |
| Hagerman RJ (1988) | 1 | 1 | 0 | 2 | Poor |
|  |  |  |  |  |  |
|  |  |  |  |  |  |
| Varley CK (1982) | 1 | 1 | 0 | 2 | Poor |
|  |  |  |  |  |  |

**Supplementary Table 4.** Summary of main adverse event in the included studies in the current meta-analysis

| Study (year) | Treatment | D/O (%) | Top reason for D/O | D/O  (AE %) | Most common adverse events | Serious AE |
| --- | --- | --- | --- | --- | --- | --- |
| Simonoff E  (2013) | MPH-IR 0.5-1.5 mg/kg/d  Placebo | 21.3  13.1 | AE  Poor adherence | 8.1  0 | Sleep difficulty (21%)^﹡^, poor appetite (15%)^﹡^  Repetitive behaviour (7%), look sad (5%) | Nil  Nil |
| Handen BL  (1999) | MPH 0.6 mg/kg/d  MPH 1.2 mg/kg/d  Placebo | 9.1  9.1  0 | AE  AE  Nil | 9.1  9.1  0 | Staring (63.6%), Dull, Not alert (54.5%), Social withdrawal (54.5%)  Dull, not alert (63.6%), Poor appetite (54.5%), Social withdrawal (45.4%)  Restless (81.8%), Excessive talking (63.6), Staring (54.5%) | Nil  Nil  Nil |
| Aman MG  (1997) | MPH 0.4 mg/kg/day Placebo | 3.3  3.3 | Behavioral deterioration  AE | 0  3.3 | Nil  Nil | Nil  Nil |
| Aman MG (1993) | MPH 0.4 mg/kg/d  Placebo | Nil  Nil | Nil  Nil | Nil  Nil | Anorexia  Nil | Nil  Nil |
| Handen BL (1992) | MPH 0.6 mg/kg/day  MPH 1.2 mg/kg/day Placebo | Nil  Nil  Nil | Nil  Nil  Nil | Nil  Nil  Nil | Nil  Nil  Nil | Nil  Nil  Nil |
| Handen BL (1990) | MPH 0.6 mg/kg/day  MPH 1.2 mg/kg/day Placebo | 8.3  8.3  Nil | AE  AE  Nil | Nil  Nil  Nil | Social withdrawal, drowsiness, staring  Social withdrawal, drowsiness, staring  Nil | Nil  Nil  Nil |
| Hagerman RJ (1988) | MPH 0.6 mg/kg/day Placebo | Nil  Nil | Nil  Nil | Nil  Nil | Nil  Nil | Nil  Nil |
| Varley CK  (1982) | MPH 0.3 mg/kg/day  MPH 0.6 mg/kg/day Placebo | Nil  Nil  Nil | Nil  Nil  Nil | Nil  Nil  Nil | Nil  Nause, decrease in appetite, difficulty sleeping  Nil | Nil  Nil  Nil |

Abbreviations: AE: adverse events; d: day; D/O: drop-out; D/O (AE %): drop-out due to adverse events; MPH methylphenidate; MPH-IR Immediate-release methylphenidate,

^﹡^Statistically significant

^﹡﹡^Not statistically significant
